# Supplementary material for: Intranasal oxytocin enhances intrinsic corticostriatal functional connectivity in women
Source: Transl Psychiatry. 2017 Apr 18;7(4):e1099–. doi: 10.1038/tp.2017.72 (PMC5416709; doi:10.1038/tp.2017.72)
Supplement: Supplementary Information [file tp201772x1.docx]

# Supplementary information

## Procedure and participants

Participants were recruited via online advertisements, the Cambridge Psychology database, local newspaper advertisements and posters spread across Cambridge. None of our participant had a diagnosis of autism or Asperger or related disorder and all participants were of Caucasian origin. Participants were instructed to abstain from alcohol and caffeine on the day of testing and from food and drink, except water, for 2 hours before spray administration. On arrival all participants were informed about the nature of the study and were given the opportunity to ask any questions for clarification. Written informed consent was obtained from all participants. Prior to the first session participants completed the Autism Spectrum Quotient (AQ) ^1^ and the Empathy Quotient (EQ) ^2^. At the start of the first session, participants were administered the Wechsler Abbreviated Scale of Intelligence (WASI; ^3^) and National Adult Reading Test (NART; ^4^). Descriptive statistics on these measures are provided in table S1.

Before administration of the nasal spray a medical examination including measurements of blood pressure, heart rate, general wellbeing and short medical history was conducted by a GMC registered medical doctor. A pregnancy test was also administered to avoid any potential effects of OXT. A medical professional provided a prescription and remained in attendance to manage any potential adverse effects from the spray. The participants received either the active or placebo nasal spray (three puffs per nostril, alternating between sides 4IU, 6.72 µg each). Heart rate and blood pressure were also continually monitored during scanning. One participant reported feeling slightly light-headed after the first MRI session. Post-hoc un-blinding revealed that this occurred during the placebo condition. No other side effects were reported. After administration of the nasal spray participants were prepared for MRI scanning and the anatomical MRI commenced approximately 30 minutes after administration, followed by the resting-state sequence 40 minutes after oxytocin administration. This study was part of a larger project and subjects subsequently (e.g. the resting-state was always acquired first) performed 3 short computerized tasks in the scanner that are not reported here. After scanning participants were asked to judge in which order they thought they had received the sprays. None of the participants were confident in their judgment and when pressed 11 out of 25 guessed correctly.

## Image processing

Multi-echo functional images were pre-processed and denoised using the AFNI integrated multi-echo independent component analysis (ME-ICA, meica.py v3, beta1; [http://afni.nimh.nih.gov](http://afni.nimh.nih.gov/)) pipeline ^5^. This pipeline included: skull-stripping of the anatomical MPRAGE image and warping it to the MNI anatomical template, co-registration of the first TE functional data to compute motion correction and for anatomical-functional co-registration, deobliquing of functional data, 12-paramater affine anatomical-functional co-registration using the local Pearson correlation and T2* weights (lp-t2s) cost function. Each TE functional dataset was slice-time corrected and spatially aligned through application of the anatomically derived alignment matrix using nonlinear warping to MNI space (MNI152 template) with AFNI 3dQwarp. No temporal filtering or smoothing was applied to the data.

Next, functional data were decomposed into independent components (ICs) as part of the ME-ICA pipeline. Subsequently, ICs were categorized as BOLD or non-BOLD based on their weightings measured by Kappa and Rho values, respectively. Because BOLD signal changes are linearly dependent on echo time (TE), a characteristic of the T2* decay, TE dependence of BOLD signal is used to dissociate BOLD from non-BOLD signal using the pseudo-*F*-statistic, Kappa. ICs that scale strongly with TE have high Kappa scores ^5^. Conversely, non-BOLD ICs are identified by TE independence measured by the pseudo-*F*-statistic, Rho. By removing non-BOLD ICs, data are denoised for motion, physiological and scanner artefacts in a robust manner based on physical principles ^5,6^. One session for one subject had to be excluded due to technical difficulties in the realignment procedure. This subject was removed from subsequent analyses.

## Between-Component Connectivity Analysis

Time courses for each component and subject were used to model between-component connectivity during placebo and oxytocin administration. This was achieved by constructing a correlation matrix of the 22 non-noise components for each subject. Connectivity strength in this correlation matrix was measured as the correlation coefficient after running robust regression ^7^ (<https://github.com/canlab/RobustToolbox>), to mitigate bias from outlier time points. We then tested for difference in connectivity strength for placebo versus oxytocin with a paired-sample t-test for each between-component connection. Correction for multiple comparisons was achieved via Bonferroni correction at a family-wise error rate of 5%. For component pairs that survived multiple comparison correction, we computed a difference score between oxytocin and placebo on the Fisher z-transformed correlation statistics ^8^. This difference score indicates the size of oxytocin-related connectivity enhancement, with larger scores indicating larger enhancement of connectivity from oxytocin, whereas scores near 0 indicate no difference in connectivity between oxytocin and placebo. To report an effect size for any oxytocin-related effects on connectivity, we computed effect size as the mean of the difference score divided by the standard deviation of the difference score. This effect size is analogous to Cohen’s d and indicates the magnitude of effect above a null effect of 0 in units of standard deviation. To get an indication of how variable such an effect size estimate is, we used bootstrapping (1 million resamples) to identify the 95% bias-accelerated bootstrap confidence intervals around our actual effect size estimate. We also used this difference score to test for association with autistic traits as measured by the AQ using robust regression. In past work we have observed that oxytocin tends to have larger effects on individuals have higher levels of autistic traits ^9^. Therefore, we made the directional prediction that oxytocin-related effects on connectivity would be positively correlated with autistic traits.

***Table S1: Descriptive statistics on participant sample from administration study***

This table show the mean, standard deviations and range of the questionnaire scores obtained during this study. Normality was assessed using the Shapiro-Wilk test of normality and indicated that the assumption against normality should be rejected, in subsequent test we thus used parametric tests (i.e. to assess the correlation of our main outcome measure with AQ).

|  | **Mean** | **Standard  Deviations** | **Range** | | **Normality** | |
| --- | --- | --- | --- | --- | --- | --- |
|  |  |  | Min | Max | W | *p* |
| ***WASI*** | 115.3 | 13.19 | 90 | 148 | 0.956 | 0.347 |
| ***NART*** | 118.1 | 5.04 | 107 | 127 | 0.960 | 0.411 |
| ***AQ*** | 14.4 | 7.32 | 3 | 33 | 0.963 | 0.475 |
| ***EQ*** | 55.6 | 14.53 | 19 | 77 | 0.955 | 0.330 |

***Table S2: Descriptive statistics for OXTR expression in females***

Descriptive statistics for all female data from the GTEx and BrainSpan datasets. RNAseq data was summarized to RPKM and all descriptives and inferential statistics are based on these RPKM values. Two different one-sample t-tests were performed to compare OXTR expression to 0 and to non-brain (skin) tissue. These tests were performed within a permutation test (1000 permutations) to derive p-values.

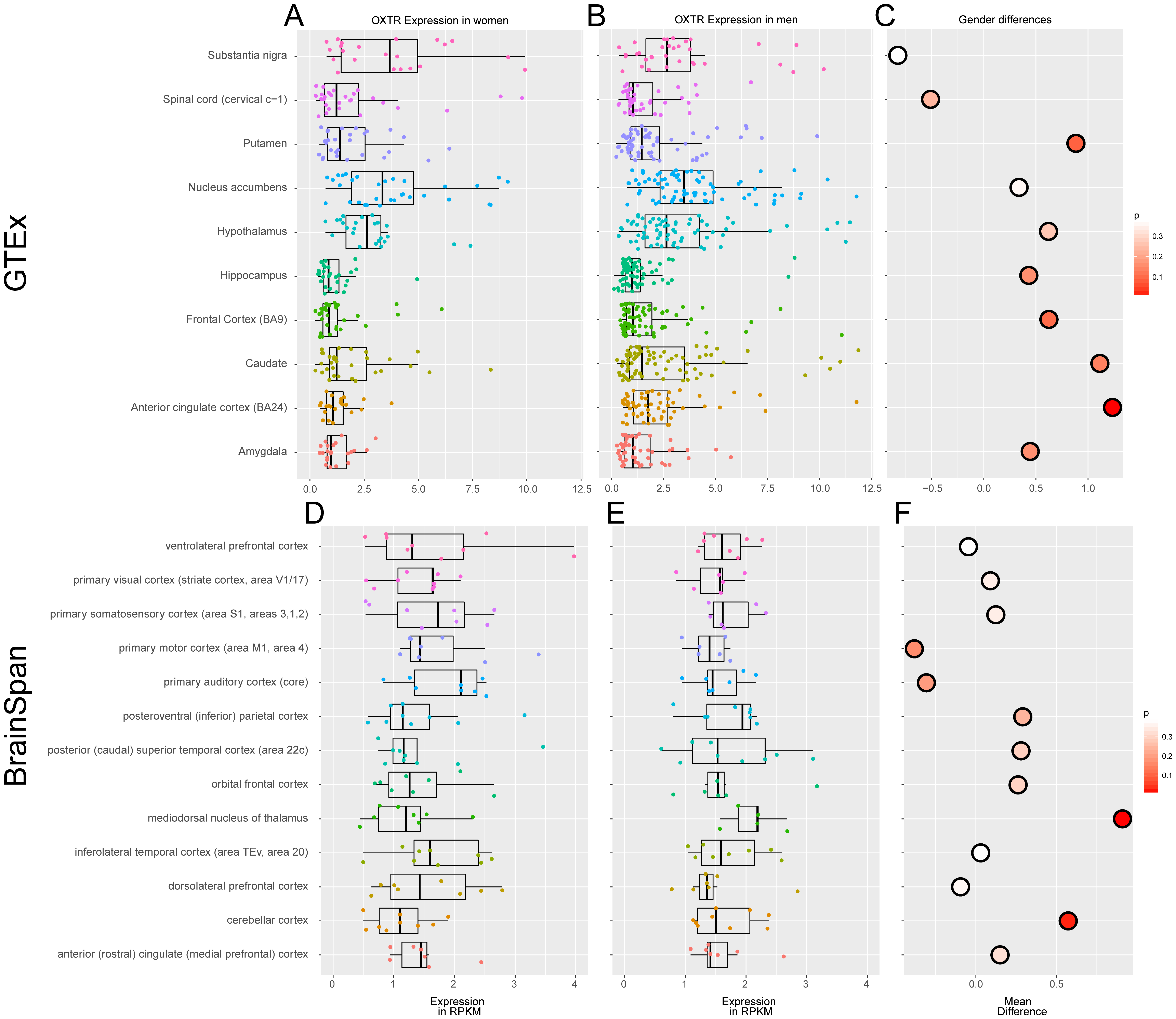


**Figure S1:** Gender comparison of OXTR expression

Although our main expression and imaging analyses focused on the oxytocin system in women, we also performed exploratory analyses on potential gender differences in OXTR expression in the same gene expression data-sets. Panels **A** and **D** show the RPKM expression values of OXTR for women, panels **B** and **E** show expression in the same brain regions for men. Panels **C** and **F** show the gender differences. Only the anterior cingulate, medial nucleus of the thalamus and the cerebellar cortex showed significant gender differences (p<0.05). In all cases where there was a significant difference men showed slightly higher expression on average. These findings should be considered exploratory as the samples are not matched, the BrainSpan datasets is limited in terms of sample size and there is in general a lot of variability in the expression of the oxytocin receptor.


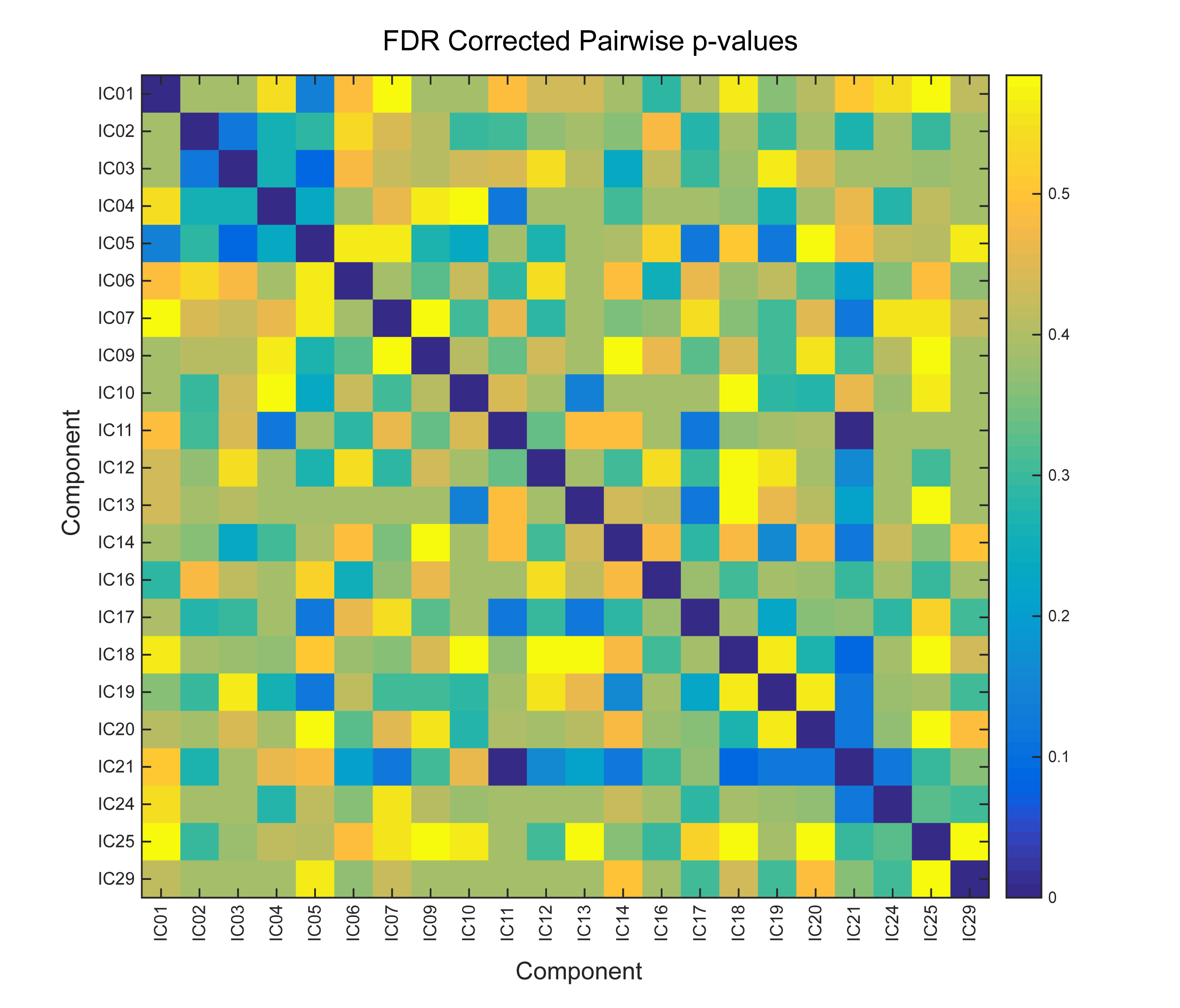


**Figure S2:** FDR corrected pairwise comparisons of all components

Only the component pair IC11-IC21 survived multiple comparison corrections, none of the other pairs were significant at p < 0.05.


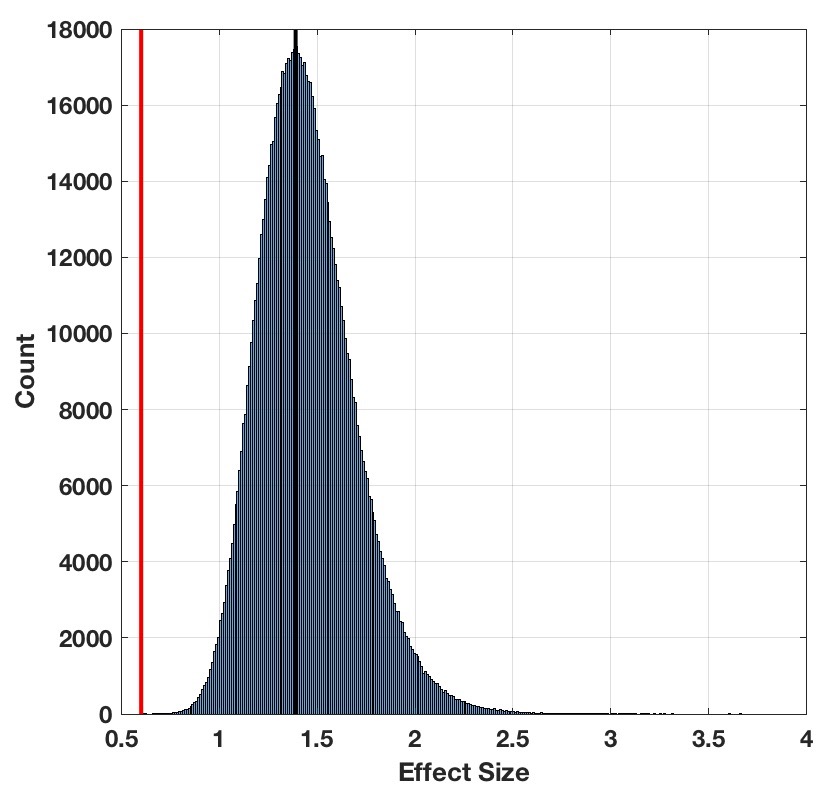


**Figure S3:** Bootstrap effect size distribution for IC11-IC21 effect.

Shows the distribution of effect size estimates calculated after bootstrapping (1 million resamples). The actual estimate is shown with the black vertical line. The red line shows the minimum effect size (d = 0.6) for achieving 80% power at an alpha of 0.05 and n=24.


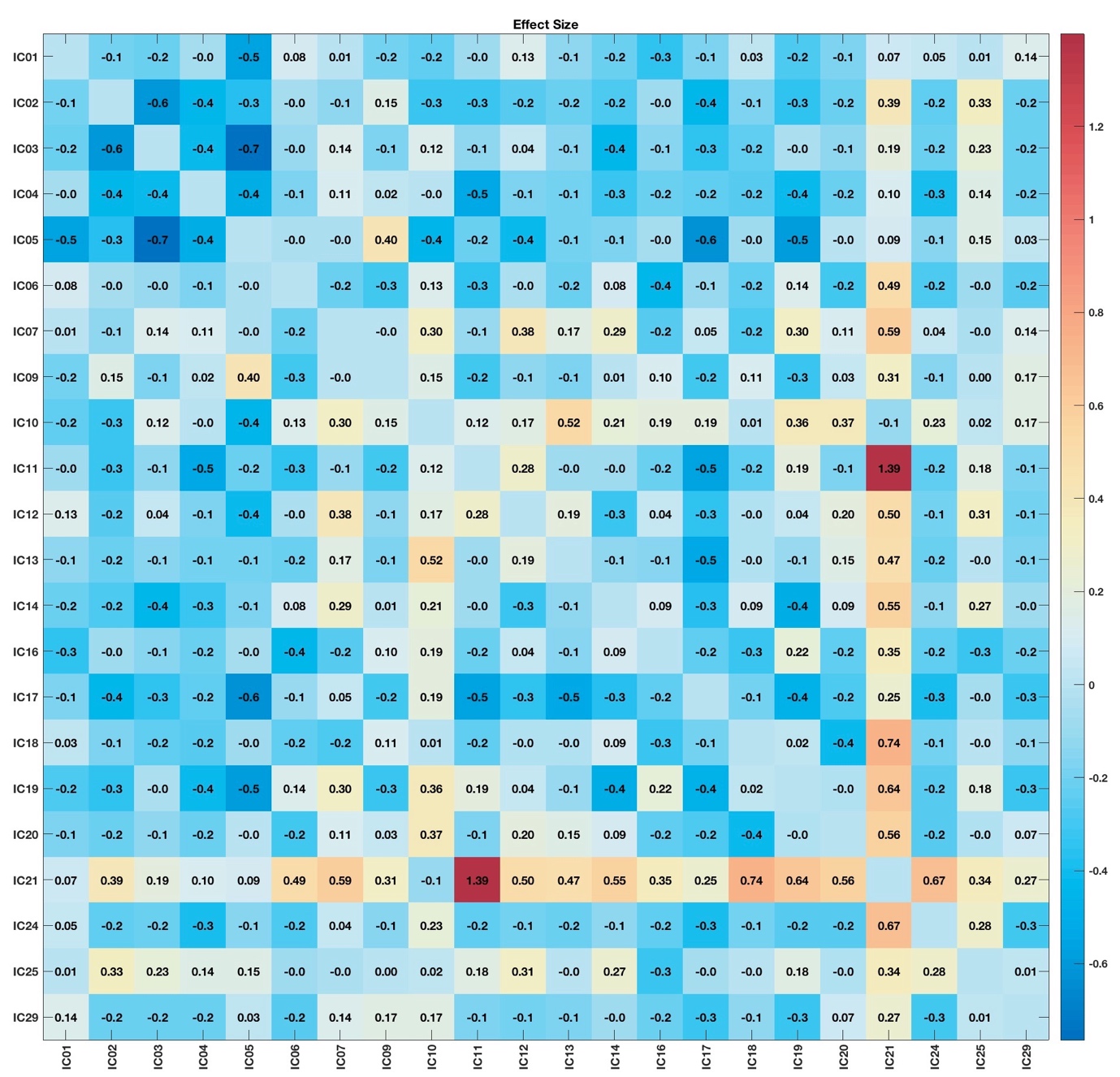


**Figure S4:** Effect size estimates for all pairwise comparisons.

Effect size is computed as the mean of the difference score (e.g., oxytocin – placebo) divided by the standard deviation of the difference score. This effect size is analogous to Cohen’s d and indicates the magnitude of effect above a null effect of 0 in units of standard deviation.

## Supplementary references

1 Baron-Cohen S, Wheelwright S, Skinner R, Martin J, Clubley E. The autism-spectrum quotient (AQ): evidence from Asperger syndrome/high-functioning autism, males and females, scientists and mathematicians. *J Autism Dev Disord* 2001; **31**: 5–17.

2 Baron-Cohen S, Wheelwright S. The Empathy Quotient: An Investigation of Adults with Asperger Syndrome or High Functioning Autism, and Normal Sex Differences. *J Autism Dev Disord* 2004; **34**: 163–175.

3 Wechsler D. *Wechsler Abbreviated Scale of Intelligence.* The Psychological Corporation: Harcourt Brace & Company.: New York, NY, 1999.

4 Nelson H, Wilson J. *National Adult Reading Test (NART)*. NFER-Nelson: Windsor, UK, 1991.

5 Kundu P, Brenowitz ND, Voon V, Worbe Y, Vértes PE, Inati SJ *et al.* Integrated strategy for improving functional connectivity mapping using multiecho fMRI. *Proc Natl Acad Sci Unites States Am* 2013; **110**: 16187–16192.

6 Evans JW, Kundu P, Horovitz SG, Bandettini PA. Separating slow BOLD from non-BOLD baseline drifts using multi-echo fMRI. *Neuroimage* 2015; **105**: 189–197.

7 Wager TD, Keller MC, Lacey SC, Jonides J. Increased sensitivity in neuroimaging analyses using robust regression. *Neuroimage* 2005; **26**: 99–113.

8 Steiger JH. Test for comparing elements of a correlation matrix. *Psychol Bull* 1980; **87**: 245–251.

9 Auyeung B, Lombardo M V, Heinrichs M, Chakrabarti B, Sule A, Deakin JB *et al.* Oxytocin increases eye contact during a real-time, naturalistic social interaction in males with and without autism. *Transl Psychiatry* 2015; **5**: e507.
